# Somatic genome architecture and molecular evolution are decoupled in “young” linage-specific gene families in ciliates

**DOI:** 10.1371/journal.pone.0291688

**Published:** 2024-01-25

**Authors:** Xyrus X. Maurer-Alcalá, Auden Cote-L’Heureux, Sergei L. Kosakovsky Pond, Laura A. Katz

**Affiliations:** 1 Institute of Cell Biology, University of Bern, Bern, Switzerland; 2 Department of Invertebrate Zoology, American Museum of Natural History, New York, New York, United States of America; 3 Department of Biological Sciences, Smith College, Northampton, Massachusetts, United States of America; 4 Institute for Genomics and Evolutionary Medicine, Temple University, Philadelphia, Pennsylvania, United States of America; 5 Program in Organismic and Evolutionary Biology, University of Massachusetts Amherst, Amherst, Massachusetts, United States of America; Montana State University Bozeman, UNITED STATES

## Abstract

The evolution of lineage-specific gene families remains poorly studied across the eukaryotic tree of life, with most analyses focusing on the recent evolution of *de novo* genes in model species. Here we explore the origins of lineage-specific genes in ciliates, a ~1 billion year old clade of microeukaryotes that are defined by their division of somatic and germline functions into distinct nuclei. Previous analyses on conserved gene families have shown the effect of ciliates’ unusual genome architecture on gene family evolution: extensive genome processing–the generation of thousands of gene-sized somatic chromosomes from canonical germline chromosomes–is associated with larger and more diverse gene families. To further study the relationship between ciliate genome architecture and gene family evolution, we analyzed lineage specific gene families from a set of 46 transcriptomes and 12 genomes representing x species from eight ciliate classes. We assess how the evolution lineage-specific gene families occurs among four groups of ciliates: extensive fragmenters with gene-size somatic chromosomes, non-extensive fragmenters with “large’’ multi-gene somatic chromosomes, Heterotrichea with highly polyploid somatic genomes and Karyorelictea with ‘paradiploid’ somatic genomes. Our analyses demonstrate that: 1) most lineage-specific gene families are found at shallow taxonomic scales; 2) extensive genome processing (*i*.*e*., gene unscrambling) during development likely influences the size and number of young lineage-specific gene families; and 3) the influence of somatic genome architecture on molecular evolution is increasingly apparent in older gene families. Altogether, these data highlight the influences of genome architecture on the evolution of lineage-specific gene families in eukaryotes.

## Introduction

The evolution of “young” lineage-specific genes and their origin remains poorly resolved across the eukaryotic tree of life, as most examples are limited to few model taxa (*e*.*g*., *Drosophila* and yeasts; [1, 2]), which likely skews our understanding of the evolutionary history of lineage-specific genes. Most examples of young genes arise through paralogous expansions of existing genes [3, 4]. Detection of *de novo* genes relies largely on high-quality draft genomes of closely related species to aid in pinpointing the transition of non-protein-coding segments of DNA into actively transcribed protein-encoding open reading frames [5]. However, discerning the *de novo* origins of “young” lineage-specific genes remains challenging, as examples of *de novo* genes can reflect failure in detecting homologs from close relatives, particularly for rapidly evolving proteins [6]. Given recent advances in single-cell omics techniques, there is increasingly ample opportunity to explore the evolution of lineage-specific genes in groups of microbial eukaryotes that remain largely uncultivable.

Ciliates are an ancient ~1 Gya [7] group of microbial eukaryotes, defined by the presence of distinct somatic and germline genomes in dimorphic nuclei residing in the same cell. While germline micronuclei remain quiescent outside of their sexual phases, ciliate somatic macronuclei are highly transcribed throughout their life histories and possess atypical genome architectures. Unlike the germline chromosomes, which appear more conventional (large megabase length, with centromeres and mobile genetic elements), ciliates’ somatic chromosomes are often gene-dense, lack centromeres, and are hyperpolyploid; somatic ploidy varies substantially among ciliates, ranging from ~45N in *Tetrahymena thermophila* to ~800N in *Paramecium tetraurelia* to ~15,000N in *Stylonychia lemnae* [8–10]. Additionally, there are striking differences in somatic genome architecture among ciliates as some lineages (*e*.*g*., the class Spirotrichea) extensively fragment their somatic genomes into thousands of unique gene-sized chromosomes, which are then amplified to variable copy numbers (*e*.*g*., *Chilodonella uncinata*, *Stylonychia lemnae*, *Oxytricha trifallax)* [11, 12].

Data on germline genome architecture are sparse. This is due to a variety of features including the uncultivability of most ciliate lineages and the fact that germline genomes in some clades are marked by “scrambled” regions, whereby consecutive somatic sequences are found in non-consecutive order and/or encoded on both strands of DNA in the germline (*e*.*g*., *Chilodonella uncinata* cl: Phyllopharyngea, *Oxytricha trifallax* cl: Spirotrichea, *Loxodes* sp. cl: Karyorelictea) [13–15]. These unusual patterns of genomic organization are largely attributed to duplication and decay, and have been linked to patterns of alternative processing, a DNA-based process analogous to alternative exon splicing in transcription, during the formation of a new somatic genome [13, 16, 17]. Germline scrambling itself may have evolved independently multiple times, especially as broad patterns in scrambling differ across deep nodes in the ciliate phylogeny [13–15]. Yet the impact of germline genome architecture on gene family evolution remains underexplored.

Prior work has linked ciliates’ somatic genome architecture to large-scale patterns of genome family evolution, as genome processing is associated with elevated rates of evolution in conserved protein-coding genes compared to other eukaryotes [18, 19]. The influence of ciliates’ atypical somatic genome architecture is apparent among lineages as well. Prior analyses demonstrated that ciliates with extensively fragmented somatic genomes (*i*.*e*., gene-sized somatic chromosomes, such as those in *Oxytricha trifallax* and *Chilodonella uncinata*) tend to possess larger widely conserved (*i*.*e*., ancient) gene families that are comprised of more diverse paralogs than other taxa with less extensive fragmentation [19, 20]. More recent efforts have further suggested that other aspects of ciliate biology (*e*.*g*., polyploidy and ability to divide somatic nuclei) contribute to these evolutionary patterns [17, 20]. The focus of this work is to further explore the influence of ciliates’ genome biology on the evolution of young ciliate-restricted gene families.

Here we combine analyses of published somatic genomes and transcriptomes from diverse ciliates to investigate the impact of genome architecture and biology on ciliate-specific gene families. Our focal taxa come from eight classes of ciliates and include understudied lineages such as Karyorelictea, Heterotrichea, and Litostomatea. As in previous work, we rely on bioinformatic tools, including PhyloToL [21], HyPhy’s RELAX [22], GeneRax [23] and Count [24] to analyze 5,525 ciliate-specific gene families. to infer evolutionary patterns and to evaluate the relationship of these patterns with varying genome architectures.

## Materials and methods

### Transcriptomes and genomes

Accession information for the raw transcriptomic reads and genome assemblies are found in supplementary table, S1 Table. For the representative genomes, we selected the longest isoforms of protein-coding genes using custom python scripts (https://github.com/xxmalcala/Ciliate_LSGF) for downstream analyses, whereas transcriptomes were first assembled with rnaSPAdes (v3.13.1) and then went through additional curation as described below.

For each transcriptome, putative rRNA sequences were identified with Barrnap (v0.9; https://github.com/tseemann/barrnap) and removed prior to ORF calling. The largest complete ORFs were then predicted for each transcript in the rRNA “free” transcriptome, using appropriate stop codons, as stop codon usage varies widely among ciliate taxa. Additionally, only putative ORFs with at least 200 amino acids (≥600bp) were retained for analysis of ciliate specific gene families. Following identification, all putative ORFs for that transcriptome were clustered with CD-HIT-EST (v4.8.1) [25] with the following parameters: “-G 0 -c 0.97 -aS 1.00 -aL 0.005”, to filter potential allelic variation. These were then used for downstream clustering, phylogenomic methods and analyses (https://github.com/xxmalcala/Ciliate_LSGF).

### Gene family clustering and selection

All protein coding ORFs ≥ 200 amino acids (from the transcriptomes and additional whole genome taxa) were clustered into gene families using OrthoFinder2 (v2.5.4) [26] with default parameters. Following clustering, gene families were further refined by keeping protein sequences of comparable size (from 50–150% the average gene family member size) and if the proportion of proteins from ciliates was ≥ 95%. These putative ciliate-restricted gene families were then further refined through PhyloToL [21] to remove non-homologous sequences. Gene families for further analyses were those that met the following criteria: 1) composed of ≥ 5 proteins, 2) ciliates represent ≥95% of proteins, and 3) ≥ 2 ciliate genera present.

### Gene family refinement

Transcriptome-sourced ORFs from the initially filtered gene families were further refined by examining the distribution of sequences based on the relationship between GC content at four-fold degenerate sites (GC3s) and Wright’s effective number of codons (ENc) [27]. The composition (GC3 and ENc) of surviving ORFs from putative lineage specific gene families were compared to those values from a set of 200 widely conserved eukaryotic gene families that are part of the current PhyloToL pipeline [21] to determine putative misidentified ORFs. For our analyses, lineage-specific ORFs that fell within the 10-90th percentile ranges of the GC3 of the conserved GFs were retained for further analyses and refinement. Afterwards, these ORFs surviving composition-based curation from transcriptomes were compared to the non-redundant protein set from RefSeq [28] (last accessed 12–2021) using DIAMOND [29] with default parameters, to further identify putative non-ciliate sequences, likely derived from contaminant and food sources, present in the near complete data set. These cleaned LSGFs were then used for all subsequent analyses.

### Estimating patterns of molecular evolution

For the analyses of lineage-specific gene family evolution, we limited the data to gene families with ciliates from at least two different (mutually/taxonomically exclusive) categories (*e*.*g*., **N**on-**E**xtensive **F**ragmenters, **E**xtensive **F**ragmenters, **He**terotrichea, and **Ka**ryorelictea; referred to as NEF, EF, He, and Ka). For each ORF and an associated maximum likelihood phylogeny, we assigned all branches in the phylogeny to one of the four above classes (NEF, EF, HE, KA) or an “unclassified” class. Terminal branches were labeled based on species classification, and internal branches were labeled with a specific class if and only if all of the descendant branches have also been labeled with the same class (otherwise they were “unclassified”, see S1 Fig in S1 File). Given a partitioned tree, we estimated evolutionary rate distributions using unrestricted codon-based random effects models [30] in HyPhy [22] version 2.5.41. These models estimate, for each taxonomic group **G** present in the tree, the branches assigned it, the branch-site level discrete distribution of **ω** (including for the unclassified branches, treated here as nuisance parameters). **ω** is the ratio of non-synonymous to synonymous substitution rates, and is widely used to classify the type (negative, neutral, diversifying) of selective pressures operating on ORFs [31]. Armed with group-level distributions of **ω (ω**_**G**_**)**, we ran two statistical tests to infer evolutionary patterns affecting each ORF.

First, we ran a group-level RELAX test (developed in [22]) as an extension of [30]. This test infers a group level parameter, **K**_**G**_, which can be interpreted as relaxation (**K**<1) or intensification (**K**>1) of selection relative to the reference group. For each OR, we set the reference group to **EF** or **NF** (if no **EF** sequences were present) or **HE** (if neither **EF** nor **NF** were present). A group level test compares the null model (**K**_**G**_ **= 1** for every non-reference group) to the alternative model (**K**_**G**_ **= 1** are estimated separately for every non-reference group) via a nested likelihood ratio test using the asymptotic **χ**^**2**^ with **|G|-1** degrees of freedom distribution to assess significance. Individual p-values are corrected for multiple testing using the Benjamini-Hochberg FDR procedure [32]. A significant result indicates that there are differences in selective forces between some of the tested groups; importantly, the test does not identify which individual groups contribute to the differences.

Second, for each ORF and each group, we ran a test of episodic positive diversifying selection (BUSTED[S]) [30]. This test examines whether or not there is a non-zero weight assigned to **ω > 1** (positive selection) for every group **G** separately, by comparing the unrestricted random effects model to the model where **ω** is constrained to (0,1). The FDR procedure is similarly employed here to correct for multiple testing.

### Inferring age of lineage-specific gene families

The ciliate species tree used as the basis for the COUNT analyses was based on the current NCBI taxonomy. The evolutionary histories (*e*.*g*., births) of the ciliate LSGFs were inferred with Count [24] using Dollo parsimony. Given the inherent incompleteness of the transcriptome-biased dataset, Dollo parsimony, under which a gene family may be gained only once, but lost multiple times, is appropriate to exclude the overabundance of losses that would be inferred under other approaches.

### Gene tree-species tree reconciliation

The ciliate species tree used to infer the relative timing of lineage-specific gene family births (above) was used as the species tree for reconciling the 5,525 individual gene trees. To infer patterns of gene family expansion/speciation across the ciliate phylogeny, we used GeneRax [23], with the following parameters: “—rec-model UndatedDL—strategy SPR—max-spr-radius 3—per-species-rates”. While duplications, speciation events, and losses can be inferred, we excluded the losses from our assessment given the inherent incompleteness of our transcriptome-biased dataset and the lack of even a single representative taxon with a publicly available and annotated genome for the majority of the ciliate classes in this study. These events were subsequently mapped onto the ciliate species phylogeny.

## Results

### Lineage-specific gene family sizes

**Fig 1:**
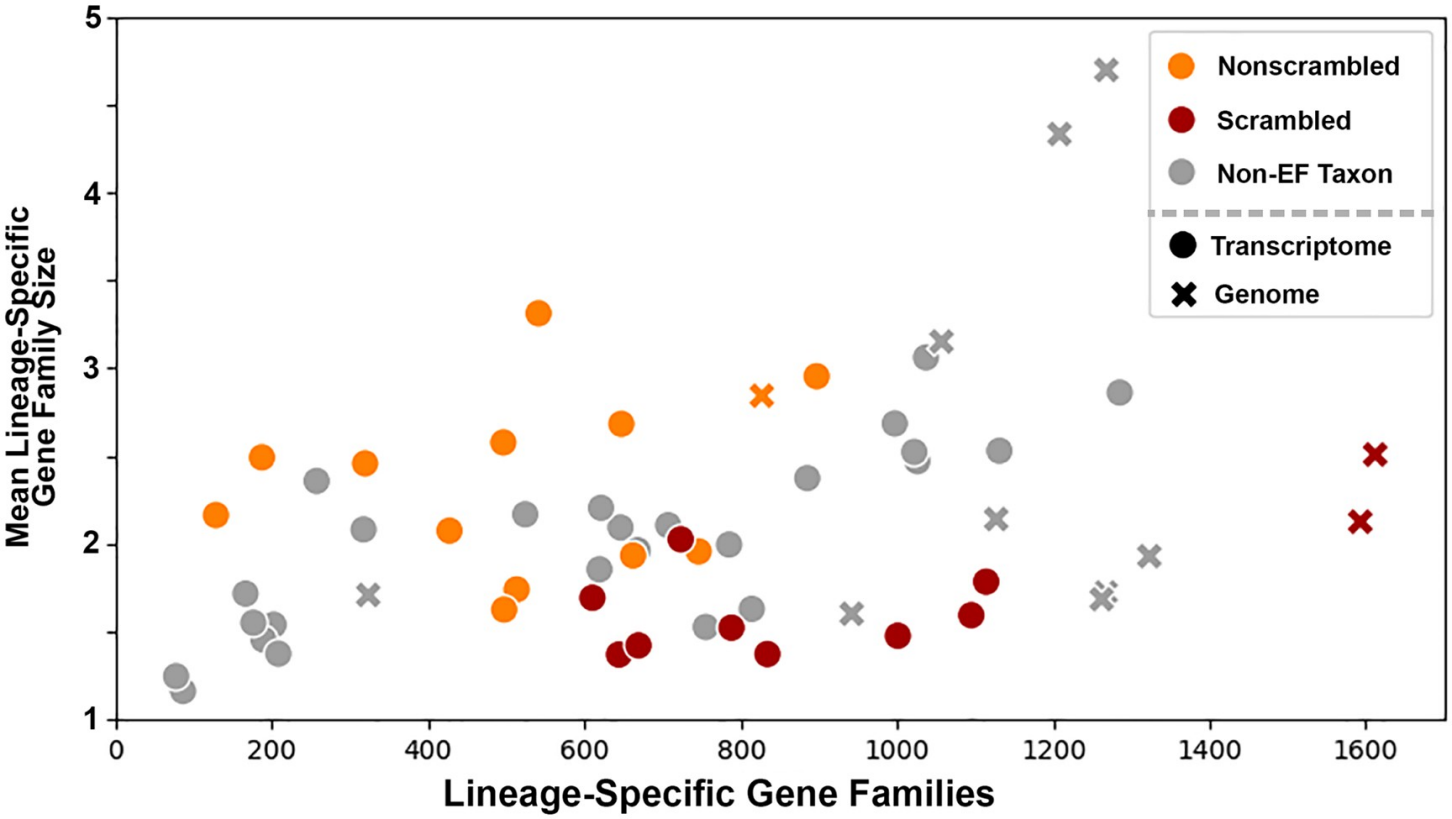
LSGF size and number reflect differences in ciliate genome architecture and data type. Overall, EF taxa with scrambled germline genomes possess more and smaller LSGFs than non-scrambled relatives. Non-extensive fragmenter taxa (*i*.*e*., He, Ka, and NEF) are shaded in gray; EF taxa with extensive germline scrambling are shown in red, whereas EF taxa without strong nor clear evidence for germline scrambling are in orange. Data source, whole genome *versus* transcriptome, does appear to impact identification of LSGFs to some degree as the highest estimates of LSGF size are found in taxa with genome sequence data.

**Fig 2:**
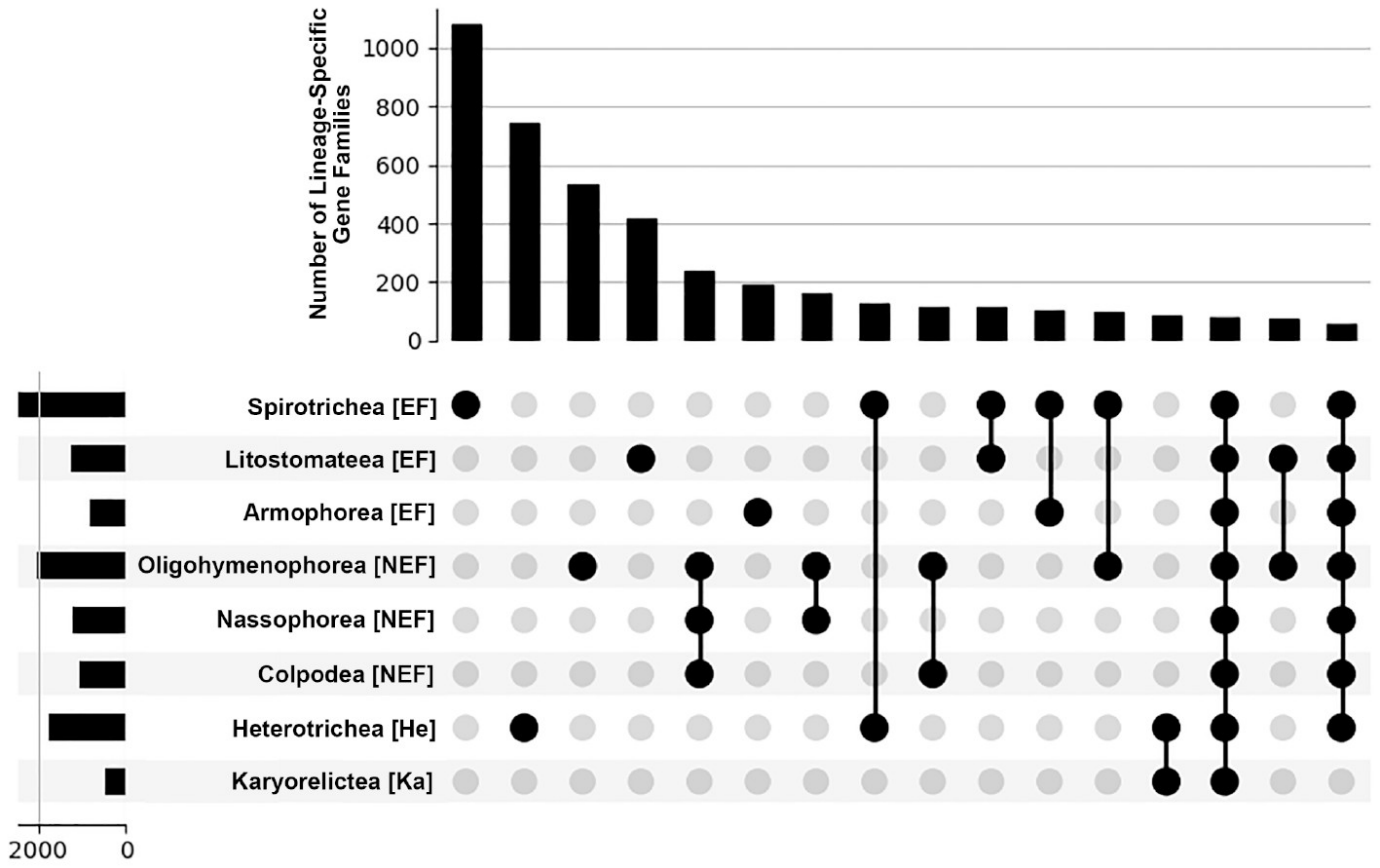
Few lineage-specific gene families are broadly shared across ciliates. Upset plot of ciliate lineage-specific gene families shows that most are limited to single ciliate classes, or lower taxonomic ranks (unconnected dots); only 57 of 5,525 LSGFs are shared across all sampled taxa (3rd column from the right).

To explore lineage specific genes families in ciliates, we compared estimates of transcript diversity per lineage-specific gene family (LSGF) for four major categories of lineages–the class **Ka**ryorelictea (Ka), the class **He**terotrichea (He), the non-monophyletic **E**xtensive **F**ragmenters (EF), and the monophyletic **N**on-**E**xtensive **F**ragmenters (NEF). Among the four major categories, the average transcript diversity of the LSGFs in Karyorelictea is the lowest, with the order of average LSGF size being: He > NF > EF > Ka (2.346, 2.124, 2.071, and 1.726, respectively; p = 0.427, One-way Anova; S2 Table). Interestingly, smaller average LSGF size may be linked to a greater number of LSGFs (Fig 1), excluding the ‘paradiploid’ Ka clade, as the EF clade harbors the greatest number of unique LSGFs (Fig 2).

Most of the LSGFs identified in this study are “young”, as only 1,673 of 5,525 LSGFs (30.28%) were shared by at least two of the four categories of ciliates, and 196 LSGFs (3.55%) possessing at least one representative taxon from all four categories (Fig 2). The lack of broadly ciliate-conserved LSGFs may represent a bias in the data type (*i*.*e*., transcriptome *versus* genome) or it may reflect a prevalence of gene loss. Disentangling these possibilities is challenging as most major clades of ciliates lack a well-annotated sequenced genome, and transcriptomic data tend to present a single life history stage (*i*.*e*., vegetative growth) for most species. This disparity is reflected in our data, as we detect a greater number of larger LSGFs in species with sequenced somatic genomes (Fig 1, S2 Table).

To evaluate the influence of data type (genome vs. transcriptomes), we further categorized patterns of LSGF size and membership at the class taxonomic rank. Here, classes with at least a single whole genome representative (Heterotrichea, Oligohymenophorea, Spirotrichea) tend to possess the greatest diversity of LSGFs (Fig 2 & S2 Fig in S1 File), with Spirotrichea (2,476) > Oligohymenophorea (2,020) > Heterotrichea (1,767). The imbalance in the number of annotated somatic genomes is strongest in the Oligohymenophorea (8/11 taxa with whole genomes), compared to the Spirotrichea (3/14) and Heterotrichrea (1/13). The imbalance in species with complete genome data in the Oligohymenophorea does impact the estimates of LSGF, which are generally greater in number and distinct from closely-related taxa with only transcriptomic representation (Fig 1). Despite the low representation of somatic genomes across the ciliate phylogeny, the mean LSGF sizes of the classes comprising the NEF clade are relatively small (Colpodea: 2.115, Oligohymenophorea: 2.186, Nassophorea: 1.957; S2 Table). Additionally, comparisons of mean and median LSGF size among related lineages in the NEF clade with (Oligohymenophorea) and without whole genome representation (Colpodea and Nassophorea) show no clear impact on LSGF size based on data-type (*i*.*e*., annotated whole genomes *versus* solely transcriptomic; p = 0.923, Kruskall-Wallis H-test). However, the inclusion of genomic datasets is more pronounced among the classes in the EF clade (Armophorea: 2.374, Litostomatea: 2.518, Spirotrichea: 1.793; S2 Table), where annotated somatic genomes are present solely in the Spirotrichea (*Euplotes*, *Oxytricha* and *Stylonychia*).

### Lineage-specific gene family expansions reflect data type and quality

**Fig 3:**
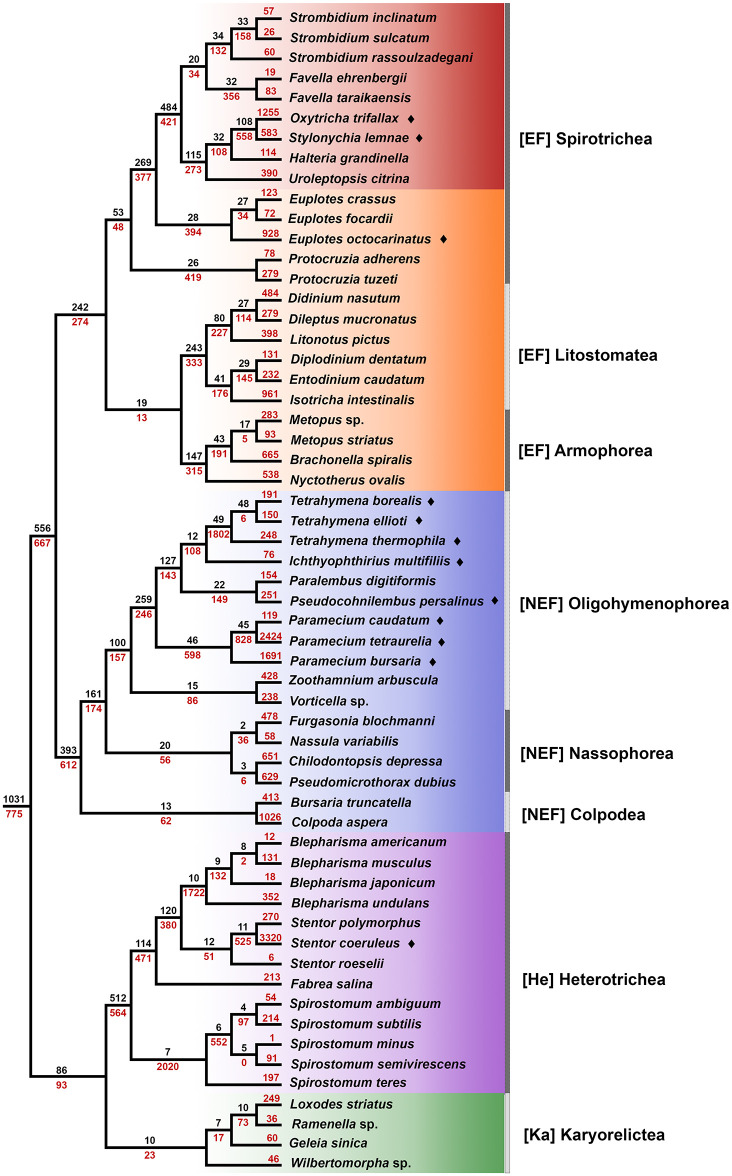
Relative age of lineage-specific gene families, but not gene duplication, in ciliates reflects genome architecture. Black values along branches represent the number of LSGF “births”, inferred through Dollo parsimony using Count [24] whereas red values represent the number of gene duplication events inferred along branches from GeneRax [23]. **EF**-clade taxa from lineages with extensive germline scrambling are highlighted in red. Whole genome taxa are marked with a diamond. Note a large number of new gene families coincide with scrambled germline genomes (EFs in red), whereas major gene duplication events are limited to clades/taxa with well annotated genomes.

Using gene tree-species tree reconciliation approaches, we also inferred the relative timing of major gene duplication events in our set of 5,525 LSGFs, noting our inability to accurately infer losses given the predominance of transcriptome data in our dataset. Similar to the shallow ages of most LSGFs, “pulses” of gene duplications are often found close to the tips, with the majority found to be species (*e*.*g*., *Oxytricha trifallax*) or genus-specific (*e*.*g*., *Spirostomum*, *Blepharisma* and *Tetrahymena*; Fig 3). Unlike our inferences of mean LSGF sizes, data type and quality does influence identification of gene duplication events. Major gene duplication events are largely limited to species and entire clades of taxa with whole genome sequences (*e*.*g*., *Tetrahymena*, *Oxytricha*, *Euplotes octocarinatus*; Fig 3) and captures well-recognized whole genome duplication events (*e*.*g*., multiple rounds of whole genome duplications in *Paramecium tetraurelia*) [33].

Although largely single-cell transcriptome based, we observe substantial gene duplication events, similar to whole genome taxa, among heterotrich ciliates. For example, 2,020 and 1,722 identifiable gene duplication events were present in the last common ancestor of *Spirostomum* species and *Blepharisma* species, respectively. These events are similar to large pulses of duplication in *Paramecium tetraurelia* and the common ancestor of *Tetrahymena* species (Fig 3). While most of these HE taxa are derived from single-cell transcriptomes, they are also substantially larger individuals (~300um to > 2mm) than most other ciliates. The large sizes of these individuals has arguably led to more comprehensive and quality transcriptome assemblies than the majority of transcriptome-based ciliate taxa in our dataset. Inferences on the relative timing of gene duplications remains difficult to ascertain, in part due to uneven phylogenetic depth of sampled lineages and the biased distribution of publicly available annotated whole genomes among ciliates.

### Germline genome scrambling is linked to LSGF diversity in extensive fragmenters

Given the diversity of estimates in LSGFs among ciliates with EF genomes, we assessed whether there was a pattern for an association between numbers and sizes of LSGFs with germline genome architectures among these ciliates. Specifically, we asked whether ciliates with scrambled germline genomes (*i*.*e*., in which somatic regions of the same gene/chromosome in the germline genome found on opposing DNA strands and/or in non-consecutive order) possessed a greater number of LSGFs, as we had previously found this pattern within the genome of the EF ciliate *Chilodonella uncinata* (Phyllopharyngea; *i*.*e*., the largest gene families come from scrambled germline loci) [14]. The potential influence of genome scrambling is apparent in the analyses, where taxa from clades with prior evidence for scrambled germline genomes have a greater number of smaller LSGFs than those with non-scrambled germline genomes (Fig 1). The mean LSGF number from spirotrich ciliates from lineages with documented germline scrambling (*e*.*g*., *Oxytricha*, *Scmidingerella*), ~1,039 LSGFs, is nearly double that of non-scrambling spirotrichs (*e*.*g*., *Euplotes*)– 634 LSGFs, as well as members of the Armophorea, ~425 LSGFs, and Litostomatea, ~559 LSGFs. We also found that the average size of LSGFs from the Spirotrichea with scrambled germlines are significantly smaller (1.799 genes *per* LSGF) compared to the non-scrambled Spirotrichea, Armophorea, and Litostomatea (2.064, 2.460, and 2.516 genes *per* LSGF respectively). A more quantitative analysis of these patterns must await full genome sequences with greater intention towards sampling of phylogenetically-diverse lineages.

The association between scrambled germline genomes and more numerous small LSGFs holds true for the few spirotrich ciliates for which we have whole genome sequencing. For example, *Euplotes octocarinatus*, a taxon without scrambling, has only 827 LSGFs with ~2.843 genes *per* family, while in distantly related lineages with scrambling–*Oxytricha trifallax* and *Stylonychia lemnae–*there are many more LSGFs, 1,612 and 1,593 LSGFs respectively, though with fewer genes *per* LSGF, ~2.508 and ~2.126, respectively. Such data are consistent with the idea that germline genome architecture contributes to patterns of origin and diversification of lineage specific genes.

### Somatic and germline genome architectures are linked to LSGF age and patterns of selection

To infer the evolutionary age of LSGFs across the ciliate phylogeny, we examined patterns of presence-absence of each of the major categories in this study (EF, NEF, He, Ka). Additionally, we explored these patterns at lower taxonomic ranks as well as through a Dollo parsimony approach employed by COUNT [24], focusing on the timing of LSGF gains (Fig 3). Given the mixed sources of our dataset (*i*.*e*., whole genome sequence, population and single-cell transcriptomics), we chose to solely focus on LSGF gains as the inference of LSGF losses is likely to be overestimated given the inability of transcriptomics to capture entire gene families (*i*.*e*., we will have missed lowly-expressed members of gene families).

The distribution of LSGFs and their gains is biased towards class-level origins as 69.72% (3,852/5,525) of all LSGFs are found in a single class of ciliates (Figs 1–3), or even in a lower taxonomic rank. For example, the branch leading to the last common ancestor (LCA) of the Heterotrichea gained 512 LSGFs that are present in a majority of the extant taxa sampled. Additionally, the branch leading to the LCA of the NEF+EF clades gained a similar number of LSGFs (556). However, these more ancient LSGFs represent the minority of the total LSGFs we found as only 18.7% (1,031/5,525) are likely present in the LCA of ciliates; the majority of LSGFs are estimated to have emerged much more recently (Fig 3). This is particularly pronounced among the EF-clade, where many LSGF gains are inferred in the respective common ancestors of the classes Armophorea and Litostomatea, rather than their shared ancestor (Fig 3). Interestingly, among members of the Spirotrichea the largest LSGF gain is found in the common ancestor of those taxa with scrambled germline genomes, which is larger than most of the LSGF gains across the entire phylogeny (Fig 3). In contrast, the greatest LSGF gains are at deeper time scales (*e*.*g*., the LCA of the NEF clade), which further highlights the potential impact of germline genome architecture on LSGF births.

**Fig 4:**
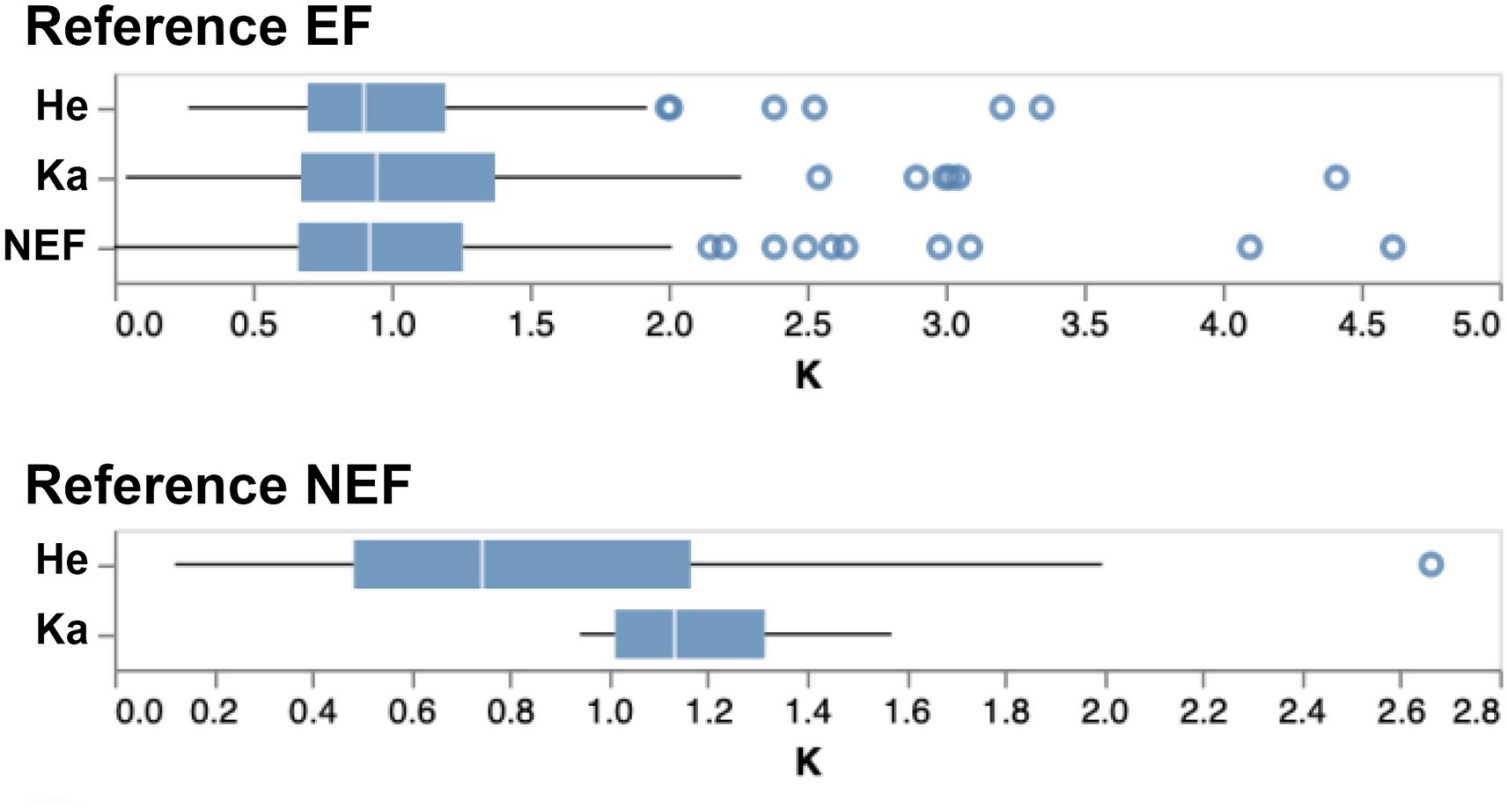
Young gene families experience greater selection intensity in taxa with extensively fragmented (EF) genomes. Distributions of estimated selection intensity (**K**) as performed by RELAX, demonstrate relaxed selection intensity (K<1) in all categories (He, Ka, NEF) relative to EFs (top). Additionally, selection intensity is greater (*i*.*e*., K>1) in Karyorelictea (Ka) and weaker (K<1) in Heterotrichea relative to the NEF category (bottom).

We estimated the relative strength of selection acting on LSGFs among our four focal clades using an extension of the RELAX method [34]. Branches in the phylogenetic tree are split into two or more groups based on the four categories (**EF**, **NEF**, **HF**, or **KA**), and one group (usually the largest, *i*.*e*., EF) is designated as reference. For each non-reference group, RELAX estimates a selection intensity parameter, **K**, where **K>1** implies that selection is intensified relative to the reference, and **K < 1**—that selection is relaxed. The null model (all **K = 1**) is tested against the alternative (some **K ≠ 1**) and a p-value is derived (see Methods). For 1,414 testable (>1 group is present with >1 branch per group) LSGFs, we found evidence for differences in selective pressures between some (or all) groups at q ≤ 0.1 in 432 (31.2%) of cases. For a further restricted set of 224/432 ORFs (15.8% of total testable alignments) where the RELAX model was deemed a good fit to the data compared to the partitioned descriptive model of RELAX (see *Methods*), we found that: compared to the **EF** reference (209/224 alignments), the other three groups tend to have relaxed selection (K<1), and compared the **NEF** reference (15 alignments), selection on **HE** tended to be relaxed while selection on **KA** tended to be intensified (Fig 4). Though the RELAX test does not directly rank groups and there are different patterns of selection found among individual ORFs, EF ciliates tend to experience the greatest selection intensity (S4 Table).

Because RELAX tests do not directly address the question of positive selection on a group of ciliates, we performed additional BUSTED[S] tests that screen for evidence of episodic diversifying selection (EDS) for each LSGF and group. As the power of EDS tests depends on the number of branches being tested (and other factors, such as divergence levels), we binned all LSFGs based on how many branches were labeled for any given group (increments of 5) and compared detection rates among the four groups (S3 Fig in S1 File). All four groups showed higher rates of EDS for increasing numbers of branches. For a fixed bin of group sizes, the **EF** lineages had the greatest rates of EDS and the general trend was **EF ≥ NF > HE ~ KA** (Table 1 & S5 Table). EDS in younger LSGFs in EFs and NEFs show no strong bias towards either category of ciliates (S5 Table).

**Table 1:** Extensive fragmenter LSGFs tend to experience more episodic diversifying selection than other groups as evidenced by greater proportion of positively selected branches as compared to other groups.

Group	Genes	Mean # Branches	Positively Selected
EF	220	14.4	54 (0.245)
NF	206	14.6	32 (0.155)
HE	232	14.4	30 (0.129)
KA	25	12.2	3 (01.20)

## Discussion

Prior work on broadly conserved eukaryotic gene families in ciliates has demonstrated that rates of molecular evolution and gene family expansion of conserved gene families (i.e those that predate the origin of ciliates) correspond to their somatic genome architecture [19, 20]. Specifically, ciliates with gene-sized chromosomes (EF clade) possess significantly larger gene families that experience more relaxed selection compared to those with large multi-gene chromosomes (NEF, He, and Ka clades) [20]. To assess if this observed relationship is apparent in lineage-specific gene families (LSGFs), we employ a conservative approach to assessing ciliate lineage-specific genes, analyzing a diverse set of ciliates by including both transcriptomic and genomic data. From this, we demonstrate that 1) most detectable lineage specific genes are young and not shared broadly across ciliate classes (Fig 1), 2) elevated rates of LSGF evolution resemble patterns from diverse model taxa (Figs 2 and 3), and 3) patterns of LSGF births and size may be attributable to germline genome architecture and unscrambling.

Ciliates are an ancient group of microbial eukaryotes, emerging >1 Gya [7, 35] and our estimates of “young” lineage-specific genes are defined as being present in at least two ciliate genera. This places the minimum age of most LSGFs to ≥100 Mya as the origins of model ciliate genera such as *Tetrahymena* and *Oxytricha* are estimated to have evolved ~250 Mya and ~100 Mya, respectively [35]. Regardless, the overwhelming majority of LSGFs that we detected are relatively young as only 30.64% (1,693/5,525) are shared among taxa from at least two of the major categories of somatic genome architecture (*e*.*g*., EF and NEF; Fig 1). Among eukaryotes for which whole genome annotations are widely available (*e*.*g*., metazoans, fungi, plants), LSGF gains and losses have pronounced tempos, with bursts of gene family births occurring at the “extrema” (very early and often very recently), and are often attributed to major group or species-specific “innovations” [36–38].

Despite the disparity in data type (few whole genome *versus* many transcriptome data) with our ciliate sample, the tempo of LSGF births does follow a similar trend at deep genome-architecture “defining” nodes (*e*.*g*., the emergence of **E**xtensively **F**ragmented somatic genomes; Fig 3), as only ~18.7% (1,031 of 5,525) of the LSGFs detected were likely found in the last ciliate common ancestor. Additionally, significant numbers of LSGFs characterize most well described classes of ciliates (*e*.*g*., Oligohymenophorea), with the exception of the data poor Karyorelictea. The relative absence of recent births of LSGSs is likely due to the divergence time of species analyzed here which themselves are fairly old (≥100 Mya), compared to studies of LSGFs among other eukaryotes, as consequence of our selection criteria (*i*.*e*., LSGF present in ≥2 genera).

Unfortunately, given the mixed data types and disparity in transcriptome quality across the ciliate phylogeny, we are unable to make strong interpretations on the tempo of gene duplications in LSGFs across the ciliate phylogeny. We conservatively suggest that most large-scale duplication events do reflect similar trends to LSGF births, as the bulk of duplications occur at shallow nodes (*e*.*g*., genera and species-specific). Despite the limitations inherent to working with largely transcriptomic datasets, we are able to provide additional support for several well recognized gene and whole genome duplication events in clades with numerous whole genome representatives. This includes multiple rounds of whole genome duplications in *Paramecium tetraurelia* following speciation from its last common ancestor with *P*. *caudatum* [33]. Additionally, our observations of increased gene duplication in heterotrich ciliates are superficially similar to prior work exploring somatic genome architecture on conserved gene family sizes. Without a greater abundance of whole genome representatives from this clade, these abundances are likely to reflect pronounced differences in data quality and further highlight the need for increased generation of quality somatic genomes from long understudied clades of ciliates.

We do find that the timing of LSGF emergence and diversity may reflect both the germline genome architecture and developmental processes that are well recognized in the class Spirotrichea. Most of the Spirotrich taxa in our study are from lineages with demonstrable germline genome scrambling [13, 39], a phenomenon where somatic sequences in the germline are found in non-consecutive order and/or in complementary orientations. Germline scrambling, which arises through duplication and subsequent degradation of germline loci, is known to provide a means to generate new genes, in part through alternative splicing of these duplicated loci (a DNA-based process analogous to alternative exon-splicing) [13, 14, 16, 17]. Indeed, relative to the *Euplotes* spp. that represent early diverging members of the Spirotrichea lacking widespread germline scrambling [40], ciliates with germline scrambling possess a greater number of small LSGFs (Fig 2), despite predominantly coming from transcriptomic sources. In other eukaryotes, similar pulses of LSGF births at these “intermediate” timescales (~400–500 Mya) [7, 32] are often associated with major evolutionary “innovations” (*e*.*g*., multicellularity, mating group signaling) [41], including indispensable roles in developmental processes [6, 42]. Overall, the low number of shared LSGFs among ciliates could reflect elevated rates of gene family birth in some lineages and/or rampant losses of LSGFs (neither of which we are able to distinguish between given the disproportionate number of taxa represented from single life stage transcriptomes) or are experiencing rapid rates of evolution that may contribute to homology detection failure.

Prior work on broadly conserved eukaryotic gene families (*e*.*g*., histone H4, actin) has shown elevated rates of evolution in taxa with extreme genome processing (*i*.*e*., extensive fragmentation) relative to other ciliates [19, 20]. We also found that the EF clade possessed the greatest proportion of young gene families experiencing relaxed selection. Additionally, by controlling for LSGF size, we also observe increased episodic diversifying selection among EF taxa relative to the remaining categories (Table 1). This is confounded by LSGF age as more inclusive taxonomic-rich LSGFs represent a span >800 Mya of ciliate evolution (*e*.*g*., present in He/Ka and EF/NEF). These more ancient LSGFs may be driving this pattern in a fashion similar to studies of widely conserved eukaryotic gene families in ciliates [19, 20]. Rather, the number of LSGFs with signatures of positive selection that are also solely present among the Intramacronucleata (*i*.*e*., shared between EF and NEF clades), are almost evenly split between those taxa with extreme genome processing (69 LSGFs) and those with less complex genome architectures (77 LSGFs; S5 Table). We hypothesize that the impact of somatic genome architecture on selection in evolutionarily young genes is weak at best, with young genes generally experiencing greater proportions of positive selection as described in diverse eukaryotes [42–44].

## Conclusions

These results are consistent with genome architecture as a driver of molecular evolution in ciliates. Specifically, the observations on patterns of LSGF evolution are consistent with the hypothesis that the presence of gene-size chromosomes (*i*.*e*., in EF ciliates) effectively allow for selection to operate on individual genes in the absence of gene-linkage, impacting the evolutionary rates of lineage-specific gene families. We hypothesize that germline scrambling may further contribute to the rate of gene family evolution in ciliates with gene-sized chromosomes given the duplicative nature of scrambled germline loci [17]. The generation of distinct protein-coding genes during the development of a new somatic genome through alternative processing of germline loci expands the diversity of any given gene family. Similarly, with the heightened efficacy of selection in the absence of gene linkage, the negative selective cost in “errors” during this time may be easily mitigated and/or effectively purged from the soma but note the germline [45]. Regardless, our observations further support our understanding of the influence genome architecture has on the evolution of gene families, while additionally highlighting the emergent role that the often overlooked germline genome architecture may play.

## Supporting information

S1 Table Ciliate taxa, abbreviated names, and data sources. (XLSX)

S2 Table Summary of LSGF sizes by ciliate class and genome architecture category. (DOCX)

S3 Table Summary of lineage-specific gene family membership and data type by ciliate taxon. (XLSX)

S4 Table Pairwise comparisons of point estimates of intensity parameters K. The (X,Y) entry in the table shows the number of ORFs among the 224 with significant RELAX results (q≤0.1) and good model fit, where the intensity parameters K is larger for group X than group Y (*i*.*e*., selection) in group X is intensified compared to Y. Cell (X,Y) is bolded if its value is greater than the value of the cell (Y,X) (*i*.*e*., selection) in group X is more frequently intensified relative to Y, compared to the opposite scenario.
(DOCX)

S5 Table Breakdown of patterns of episodic diversifying selection (EDS) in LSGFs of focal clades. “+” indicates the presence of EDS, whereas “-” denotes its absence.
(DOCX)

S1 File Additional figures, including exemplar tree labeling strategy for selection analyses, average LSGF size for each taxon by genome architecture category, and the summary of ORFs under selection by genome architecture category. (DOCX)
