# Correction: Somatic genome architecture and molecular evolution are decoupled in “young” linage-specific gene families in ciliates

**DOI:** 10.1371/journal.pone.0315635

**Published:** 2024-12-09

**Authors:** Xyrus X. Maurer-Alcalá, Auden Cote-L’Heureux, Sergei L. Kosakovsky Pond, Laura A. Katz

The word "lineage" is misspelled in the article title. The correct title is: Somatic genome architecture and molecular evolution are decoupled in “young” lineage-specific gene families in ciliates. The correct citation is: Maurer-Alcalá XX, Cote-L’Heureux A, Kosakovsky Pond SL, Katz LA (2024) Somatic genome architecture and molecular evolution are decoupled in “young” lineage-specific gene families in ciliates. PLOS ONE 19(1): e0291688. https://doi.org/10.1371/journal.pone.0291688
