# Supplementary figures and images for: Somatic genome architecture and molecular evolution are decoupled in “young” linage-specific gene families in ciliates
Source: PLoS One. 2024 Jan 25;19(1):e0291688. doi: 10.1371/journal.pone.0291688 (PMC10810533; doi:10.1371/journal.pone.0291688)

**Table S2. Summary of LSGF sizes by ciliate class and genome architecture category.**
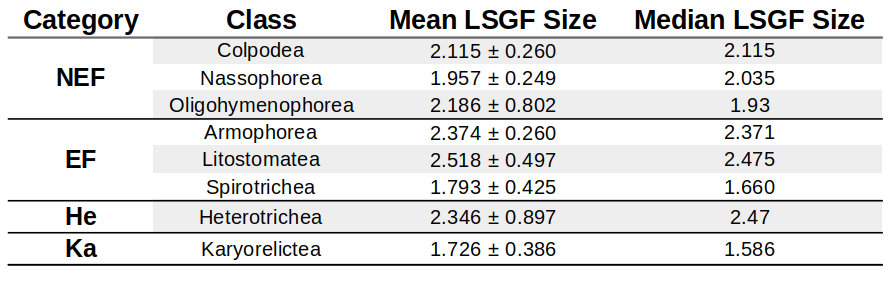

Supplement: S2 Table — (DOCX) [file pone.0291688.s002.docx]

|  | **EF** | **KA** | **NEF** | **HE** |
| --- | --- | --- | --- | --- |
| **EF** | - | 34 | 89 | 86 |
| **KA** | 31 | - | 30 | 33 |
| **NEF** | 74 | 24 | - | 63 |
| **HE** | 58 | 31 | 51 | - |


Supplement: S4 Table — The (X,Y) entry in the table shows the number of ORFs among the 224 with significant RELAX results (q≤0.1) and good model fit, where the intensity parameters K is larger for group X than group Y (i.e., selection) in group X is intensified compared to Y. Cell (X,Y) is bolded if its value is greater than the value of the cell (Y,X) (i.e., selection) in group X is more frequently intensified relative to Y, compared to the opposite scenario. (DOCX) [file pone.0291688.s004.docx]
