# Supplementary material for: Somatic genome architecture and molecular evolution are decoupled in “young” linage-specific gene families in ciliates
Source: PLoS One. 2024 Jan 25;19(1):e0291688. doi: 10.1371/journal.pone.0291688 (PMC10810533; doi:10.1371/journal.pone.0291688)
Supplement: S5 Table — “+” indicates the presence of EDS, whereas “-” denotes its absence. (DOCX) [file pone.0291688.s005.docx]

| **EF** | **NEF** | **HE** | **KA** | **LSGF Count** |
| --- | --- | --- | --- | --- |
| **-** | **+** |  |  | 64 |
| **+** | **-** |  |  | 56 |
| **+** | **-** | **-** |  | 49 |
| **-** | **+** | **-** |  | 38 |
| **+** | **-** | **-** | **-** | 32 |
| **+** | **+** | **-** | **-** | 21 |
| **-** |  | **+** |  | 21 |
| **+** |  | **-** |  | 20 |
| **-** | **+** | **-** | **-** | 18 |
| **-** | **-** | **+** |  | 18 |
| **+** | **+** | **-** |  | 17 |
| **+** | **+** |  |  | 13 |
| **-** | **-** | **+** | **-** | 13 |
| **+** | **-** | **+** |  | 12 |
| **+** | **-** | **+** | **-** | 10 |
|  | **-** | **+** |  | 10 |

**Table S5. Breakdown of patterns of episodic diversifying selection (EDS) in LSGFs of focal clades.** “+” indicates the presence of EDS, whereas “-” denotes its absence.
