# Supplementary material for: Somatic genome architecture and molecular evolution are decoupled in “young” linage-specific gene families in ciliates
Source: PLoS One. 2024 Jan 25;19(1):e0291688. doi: 10.1371/journal.pone.0291688 (PMC10810533; doi:10.1371/journal.pone.0291688)
Supplement: S1 File — (DOCX) [file pone.0291688.s006.docx]

| 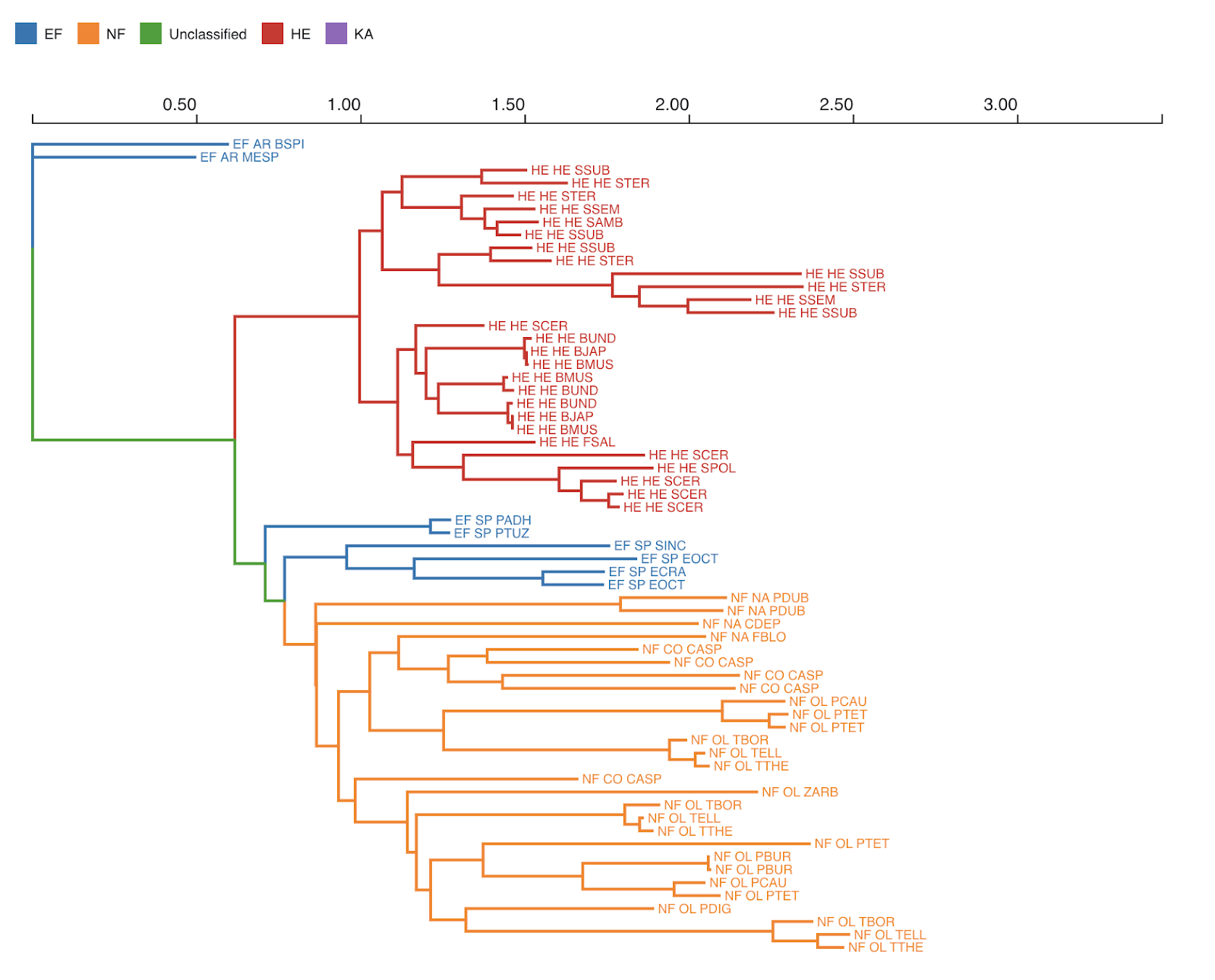 |
| --- |
| **Figure S1.** **An examplar ORF tree labeling strategy for selection analyses.** |


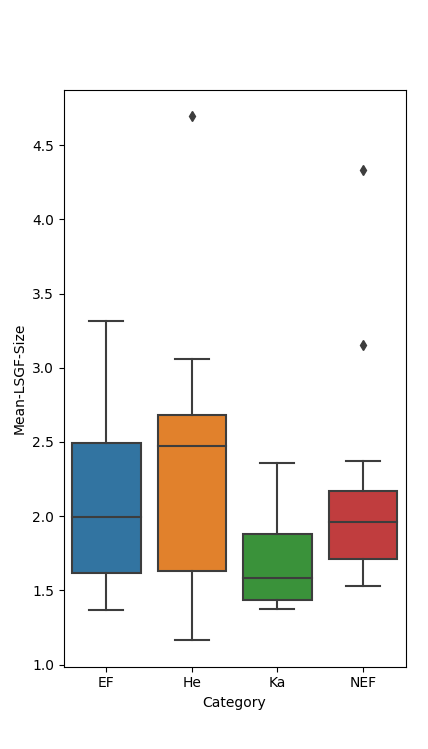
**Figure S2.** **Boxplot of average LSGF size for each taxon by respective genome architecture category.**


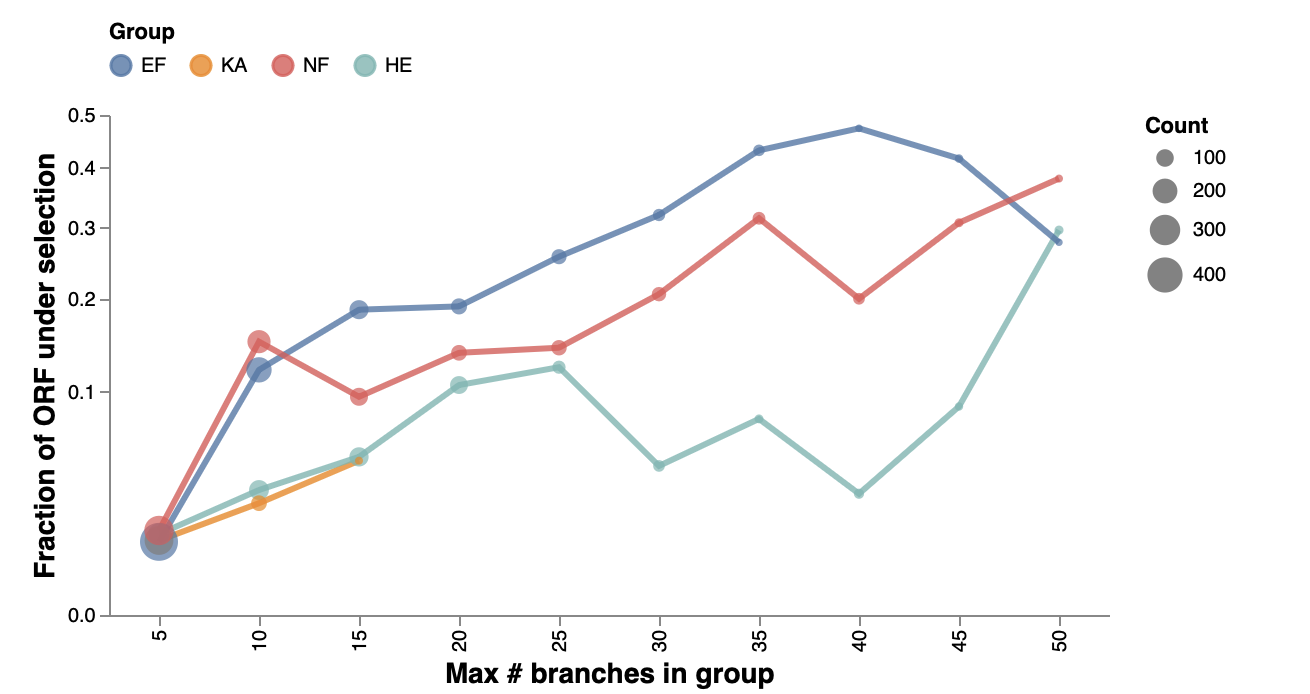


**Figure S3. Proportion of ORFs under selection compared to number of branches for each genome architecture category.** Count and point size corresponds to the number of LSGFs. Ease of detecting episodic diversifying selection is tied to the number of branches from any given group.
